# New complementary perspectives for inpatient physical function assessment: matched clinician-report and patient-report short form measures from the PROMIS adult physical function item bank

**DOI:** 10.1007/s11136-022-03089-z

**Published:** 2022-03-08

**Authors:** Michael A. Kallen, Heather E. Brown, Joeffrey R. Hatton, William A. Doyle, Ryan Murphy, Ryan Elliott, Mark A. Gutierrez, Emma L. Catherwood, Heather P. Pitman, Vincent X. Liu, Richard C. Gershon

**Affiliations:** 1 Department of Medical Social Sciences, Northwestern University Feinberg School of Medicine, Chicago, IL USA; 2 Kaiser Permanente, Kaiser Foundation Hospitals and Health Plan, Oakland, CA USA; 3 The Permanente Medical Group, Oakland, CA USA; 4 Division of Research, Kaiser Permanente, Oakland, CA USA

**Keywords:** Inpatient physical function, Physical function short form, Clinician-reported outcome, Patient-reported outcome, Performance assessment, Short form development

## Abstract

**Purpose:**

To develop two item content-matched, precise, score-level targeted inpatient physical function (PF) short form (SF) measures: one clinician-reported, one patient-reported. Items were derived from PROMIS PF bank content; scores are reported on the PROMIS PF T-score metric.

**Methods:**

The PROMIS PF item bank was reviewed for content measuring lower-level PF status (T-scores 10–50) with high item set score-level reliability (≥ 0.90). Selected patient-reported (PR) items were also edited to function as clinician-reported (CR) items. Items were reviewed by clinicians and field tested; responses were assessed for meeting PROMIS measure development standards. New CR and PR items were calibrated using patient responses to the original PROMIS PF items as anchoring data. SFs were constructed, based on content and precision.

**Results:**

Nine PROMIS PF items were candidates for CR and PR inpatient PF assessment; three new items were written to extend content coverage. An inpatient sample (*N* = 515; 55.1% female; mean age = 66.2 years) completed 12 PR items and was assessed by physical therapists (using 12 CR items). Analyses indicated item sets met expected measure development standards. Twelve new CR and three new PR items were linked to the PROMIS PF metric (raw score *r* = 0.73 and 0.90, respectively). A 5-item CR SF measure was constructed; score-level reliabilities were ≥ 0.90 for T-scores 13–45. A 5-item PR SF measure was assembled, mirroring CR SF content.

**Conclusions:**

Two item content-matched SFs have been developed for clinician and patient reporting and are an effective, efficient means of assessing inpatient PF and offer complementary perspectives.

**Supplementary Information:**

The online version contains supplementary material available at 10.1007/s11136-022-03089-z.

## Plain English summary

We wanted to develop two new inpatient physical function measures: one for clinicians to use, and one for patients. We first looked at the PROMIS Physical Function item bank to find items measuring physical function status at levels such as hospital inpatients might experience. We found nine PROMIS Physical Function items that were good candidates for clinician and patient reporting; we also wrote three new items to include additional assessment content. We then made sure items were available in versions for both clinicians and patients to use in reporting. We tested our items with a hospital inpatient sample (*N* = 515; 55.1% female; mean age = 66.2 years), and our analyses indicated that these new item sets met good, established measure development standards. We “linked” all of our new items, i.e., put them on the same measurement scale, as the items we had found and used from the PROMIS Physical Function item bank so it would be possible to use and interpret scores from the clinician measure and the patient measure in a similar way. We then picked the “best” five items, content-wise as well as reliability-wise, to make a 5-item clinician measure. For a matching 5-item patient measure, we used the patient-report items with the same item content as the clinician items. Thus, we successfully developed two item content-matching measures, each only five items in length, for clinicians and patients to use when assessing inpatient physical function status, thereby offering complementary perspectives on a patient’s physical functioning.

## Introduction

Physical function (PF) is a critical component of overall health [1, 2]. Yet, in the context of acute illness, PF is often poorly measured and tracked [3]. In addition, many patients experience new, significant disability in the course of hospitalization [4–6]. Following discharge, patients of all ages face recovery from the acute cause of their hospitalization and the trauma of hospitalization itself; many never return to their prior level of functioning [3, 7–9]. While numerous instruments measuring PF exist, few have been developed that can readily be deployed to track patients’ functional trajectory before, during, and after hospitalization [10]. In the absence of such instruments embedded across the care continuum, healthcare providers, patients, and caregivers miss an opportunity to understand and communicate about the occurrence of important PF changes. Without a common instrument tracking functional trajectories across care settings, clinical conditions, and time, it is difficult to develop patient- and population-level approaches for recognizing and mitigating decline in PF during the high risk, peri-hospitalization period.

Advanced electronic health records (EHRs) provide extraordinary capabilities to reliably store and communicate patient data within a healthcare system. However, the development and availability of PF assessment instruments, generalizable to multiple disease states and able to be administered across a variety of care settings and time periods, has been lagging [11, 12]. Patient-Reported Outcomes Measurement Information System (PROMIS) instruments offer these capabilities and have been integrated within multiple EHR systems [13, 14]. Nevertheless, existing PROMIS PF assessments are infrequently utilized during hospitalization: They may not meet unique needs of the hospital environment, such as including a PF assessment that is quick and easy to use, transparent and simple to understand, and relevant to the mobility activities and disposition planning routinely occurring in the hospital [3, 15]. Additionally, it is important that inpatient PF assessments also be conducted by clinicians. In the dynamic inpatient environment with acutely ill patients, patient reports alone may not support all treatment, safety, and disposition decision-making needs, particularly for patients with significant cognitive and/or new functional impairment or who lack insight into their current abilities and needs.

Our purpose was to develop two separate content-matched, precise, score-level targeted inpatient PF short form (SF) measures – one clinician-reported, one patient-reported – to close this gap in inpatient PF assessment. Items were derived from existing PROMIS PF bank content, and scores reported on the PROMIS PF metric.

### Study objectives

We outlined four objectives for developing clinician-report (CR) and patient-report (PR) inpatient PF SFs: (1) Review the PROMIS PF bank for PR items of appropriate content targeting the 10–50 T-score range; rewrite them as CR items; (2) field test and evaluate the proposed CR and PR items, which were administered by physical therapists (PTs); (3) link CR items to PR items on the PROMIS PF metric; (4) construct CR and PR SF measures.

## Methods

### Objective 1: identify PF items

We reviewed the PROMIS PF bank (version 2.0; *N* = 165 PR items) for items with clinically relevant content for clinician and patient (self) assessment of inpatient PF status. We focused on items targeting the 10–50 T-score range (US general population mean = 50, standard deviation (SD) = 10) providing, as a set, superior score-level reliabilities (≥ 0.90). We wanted measures that would (a) reliably assess inpatients with lower PF status at intake and (b) reliably assess clinically meaningful PF change (e.g., improvement) throughout hospitalization and at discharge. We identified candidate PR items, which we also rewrote into candidate CR items, for clinician completion following direct inpatient observation.

### Objective 2: field test and evaluate items

We field tested CR and PR items in a representative sample of KP inpatients exhibiting a range of PF status to evaluate item performance and establish preliminary evidence of their reliability and validity.

#### Sample

Inpatients were patients treated in the KP healthcare system during routine sessions with physical therapists (PTs) and represented a convenience clinical sample acquired from February 6, 2018 to September 24, 2018. There was no *a priori* effort to ensure a demographically or clinically representative patient sample was assessed; all patients receiving a PT consult were assessed until a representative sample of PF ranges and a robust sample size (*N* ≥ 500) for conducting planned analyses was achieved. Eligibility criteria included patients ≥ 18 years old, English-speaking, and receiving routine PT evaluation. Exclusion criteria were based on patient ability to provide responses, i.e., having a condition affecting response validity (e.g., dementia). CR and PR measure development was approved by KP’s IRB (study status: exempt).

#### Data elements

The demographics “age” (a continuous variable; years > 89 coded as “89” to preserve anonymity) and “gender” (female, male) were collected, as was whether assistance was required for inpatients to complete PR items [i.e., inpatients were asked “Do you need help filling out the form?” (*Yes*/*No*)].

#### Measures

Inpatients completed PR items. Inpatients’ assigned PT completed CR items.

#### Procedures

For study-eligible inpatients, their assigned PT invited them to complete PR items (paper-based self-administration). The PT, blinded to inpatient responses, then conducted the planned PT encounter (evaluation/treatment) before completing CR items. Clinician responses were identified as from a particular, yet anonymous, clinician. A fully-blinded 5-digit clinician rater identification was used, enabling study investigators to determine the number of clinicians providing ratings and average number of ratings per clinician. Inpatient PR responses were later matched with CR responses.

Our strategy was to avoid undue influence on patient self-assessment and on clinician patient assessment, uncoupling one measurement process from the other. Patient self-assessment occurred first, in isolation from clinician direct assessment. It was and remains important for patients to perform their evaluations independently: They may need to provide assessments for time periods prior to admission and post-discharge. Clinician assessment began only post patient self-assessment, with clinicians blinded to patient responses. Guided PT activities and patient observation were conducted by clinicians and exclusively part of the clinician assessment process.

#### Classical test theory (CTT) analyses

We conducted item and scale analyses of CR and PR items, obtaining summaries of response category distributions, Cronbach's alpha internal consistency reliability, and range of adjusted (corrected for overlap) item-total score correlations. Items were scored so higher scores represented higher levels of PF status.

#### Summed score distribution

We created summed total scores for CR and PR item sets. We identified minimum/maximum possible scores and displayed score distributions. We reported minimum/maximum observed scores and distribution means, SDs, skewness, excess kurtosis, and percent of cases with minimum/maximum possible scores, using ≥ 15% as the criterion for floor/ceiling effect [16, 17]. For skewness and excess kurtosis, we considered values from − 1.0 to + 1.0 to reflect distributional essential normality [18]. We calculated the Pearson correlation between CR and PR summed scores and reviewed its nature and magnitude for initial validity evidence and the appropriateness of conducting item response theory (IRT)-based item set linking. We anticipated a correlation of substantial magnitude between CR and PR scores, yet not so large (e.g., ≥ 0.90) to imply one score was redundant with respect to the other.

#### Categorical confirmatory factor analysis (CCFA)

We assessed CR and PR item set dimensionality, conducting CCFAs with a weighted least square-mean and variance adjusted (WLSMV) estimator [19]. We estimated single-factor models, using inter-item polychoric correlations, examining residual correlations for item local dependence (correlations > 0.20). We summarized results via model fit index criteria: confirmatory fit index (CFI) and Tucker–Lewis Index (TLI) ≥ 0.95, root mean square error of approximation (RMSEA) < 0.10, standardized root mean residual (SRMR) < 0.08 [20, 21]. For models not attaining fit criteria, we conducted confirmatory bifactor analyses (CBFA), diagnosing multi-dimensionality impact on fit and determining if (a) items were essentially unidimensional (omega-H index value > 0.80), and (b) the general factor, representing all items, had the majority of reliable variance attributable to it, supporting use of a total score [22]. We combined CCFA and CBFA results to establish item set unidimensionality, required for IRT modeling.

#### Differential item functioning (DIF)

We evaluated CR and PR items for DIF, investigating patient age (≤ 65 vs. > 65) and gender (female vs. male) factors. Stage 1: Using hybrid logistic ordinal regression-IRT ability scores, we flagged items for DIF (Nagelkerke pseudo-R^2^ ≥ 0.20 [23, 24]. Stage 2: We subjected flagged items to “score impact” studies, using unadjusted vs. DIF-adjusted theta scores, computing the score difference SD, root mean square difference (RMSD), and percent of cases whose unadjusted vs. DIF-adjusted score difference (absolute value) exceeded unadjusted score standard error (SE), i.e., non-trivial differences.

### Objective 3: link new CR items to PROMIS PF

We created a measurement link from CR items to PF. We calculated summed score Pearson and disattenuated correlations (corrected for unreliability) between CR and PR items; given similarities of content and assessment purpose, we anticipated inter-correlations would be ≥ 0.70, supporting IRT-based linking [25, 26]. We administered CR and PR items to a matched inpatient-inpatient’s PT sample, akin to a single-group design, enabling linking CR to PR items (existing PF items) via a reduced error design [25, 26]. Clinicians could then interpret CR scores centered on and using the PF T-score metric.

#### Item/score linking method

We followed PROsetta Stone Project linking methodology, using IRT-based “fixed parameter calibration” (FPC) to fix item parameters at established values [27–30], i.e., anchoring existing PR parameters at their PF values and calibrating only new CR items. CR parameters were then on the PROMIS PF (PR) item metric [27–30].

### Objective 4: construct SF measures

For CR and PR items, to reduce response burden, we selected representative, clinically relevant item subsets providing best attainable score-level reliabilities across our targeted T-score range. Our study team (clinicians, measurement experts) tested proposed item subsets for each SF, employing item content, item discrimination/location parameters, and score-level item/test information to optimize clinical relevance and score-level precision.

#### SF T-score distribution

We calculated T-scores for CR and PR SF measures, identifying minimum/maximum possible scores and displaying distributions. We reported minimum/maximum observed scores, distribution means, SDs, skewness, excess kurtosis, and percent of cases having minimum/maximum possible scores (floor/ceiling effects).

#### SF correlation and reliability

We calculated the Pearson correlation between SF measure scores, anticipating it to be substantially large, yet not large enough to imply score interchangeability or redundancy. We plotted CR vs. PR scores to visually identify well-aligned vs. less-well-aligned score pairs, given that each score set represented a unique source of information or perspective (clinician vs. inpatient). We constructed a histogram depicting the distribution of clinician vs. patient score differences, assessing distributional normality and typical score difference magnitude.

We reported the T-score range for which score-level reliability was ≥ 0.90, alpha reliability, and IRT SE-based reliability, the latter two representing overall, summary reliability estimates [31].

#### SF summed score to T-score conversion tables

Preferred (most precise) SF scoring employs IRT response pattern scoring: Individual responses to items are scored via “weights” derived from each item’s unique parameters. We also created scoring conversion tables, which provide the most appropriate T-score (and SE) for all possible summed scores, without requiring logistically, computationally demanding response pattern scoring.

### Sample size

Samples of *N* = 500 respondents, with item responses across the full range of response options, are recommended to produce accurate, stable graded response model (GRM)-based calibrations [32, 33]. This sample size provides better than adequate (80%) power for validity statistics, where sample sizes half this magnitude are typically sufficient (e.g., *N* = 510 inpatients provides > 90% power for correlational analyses) [34]. IRT-based DIF analyses also require a minimum *N* = 500 (with *n* ≥ 200 per subgroup) [35]. Finally, *N* = 500 is appropriate for linking analyses, for which a minimum *N* = 400 has been recommended [25, 26].

## Results

### Objective 1: identify PF items

We identified nine PROMIS PF bank items with content relevant for clinician/patient assessment of PF status (Table 1). Items targeted the 10–50 T-score range, providing reliabilities ≥ 0.90 for T-scores 13–47, attaining our goal of reliably assessing lower PF status inpatients and PF change (improvement) to approximately average PF status levels.

**Table 1:** CR and PR item content: 12-item sets

PROMIS PF PR Items	PROMIS PF CR Items
**PR Item Stem**	**Rewritten CR Item Stem**
**(> > > Rewrite > > >)**	
*Are you able to…*	*How much human assistance does the person need to…*
Turn from side to side in bed?	Turn from side to side in bed?
Sit on the edge of a bed?	Sit on the edge of a bed?
Get out of bed into a chair?	Get out of bed into a chair?
Stand up from an armless straight chair?	Stand up from an armless straight chair?
Walk a block (about 100 m) on flat ground?	Walk about 100 m on flat ground?
Climb up five steps?	Climb up five steps?
Bend down and pick up clothing from the floor?	Bend down and pick up clothing from the floor?
Stand up on tiptoes?	Stand up on tiptoes?
Squat and get up?	Squat and get up?

**Rewritten PR Item Stem**	**New CR Item Stem**
**(< < < Rewrite < < <)**	
Are you able to…	How much human assistance does the person need to…
Sit on the edge of the bed to lean forward to reach for something?	Sit on the edge of the bed to lean forward to reach for something?
Walk around the room?	Walk around the room?
Walk 50ft?	Walk 50ft?

#### CR items and new item content

We rewrote the nine candidate PR items into candidate CR items, for clinician response following direct observation. Content review by our study’s clinician experts identified coverage gaps; we wrote three new CR items (and parallel PR items) to include recommended content (Table 1).

#### Response categories

For PR items, we used their standard PF 5-point response options [*Without any difficulty* (5) to *Unable to do* (1)]. For CR items, a new, 5-point response option set, appropriate for direct inpatient observation, was developed [*None* (5) to *Total* (1)] (Table 2).

**Table 2:** CR and PR SF item content and response options

PR Inpatient PF SF	CR Inpatient PF SF
**Item Content**	**Item Content**
*Are you able to…*	*How much human assistance does the person need to…*
Turn from side to side in bed?	Turn from side to side in bed?
Sit on the edge of a bed?	Sit on the edge of a bed?
Get out of bed into a chair?	Get out of bed into a chair?
Walk around the room?	Walk around the room?
Walk a block (about 100 m) on flat ground?	Walk about 100 m on flat ground?
***Response Options (Scoring value)***	***Response Options (Scoring value)***
Without any difficulty (5)	None (5)
With a little difficulty (4)	Supervision (4)
With some difficulty (3)	A little bit (3)
With much difficulty (2)	Quite a bit (2)
Unable to do (1)	Total (1)

### Objective 2: Field test and evaluate items

#### Sample

Our inpatient field testing sample (*N* = 515) was 18–89 years old (mean = 66.2, median = 68.0, SD = 14.9); 55.1% were females; 28.3% required assistance to complete PR items. We present additional sample clinical characteristics in Table 3. Thirty-six clinicians (PTs) completed CR items per assigned study participant (mean = 14.2 assessments per clinician). PTs averaged 15.4 years (SD = 8.2) of practice experience; 69.4% were female.

**Table 3:** Inpatient sample characteristics

Patient characteristic	Descriptive statistic*
Age, years	66.4 ± 15.2
Male gender	231 (44.9)
Body mass index	30.1 ± 8.2
Length of stay, days	3.3 (1.9 – 5.9)
Admitted via Emergency Department	292 (57.9)
**First hospital unit**	
General medical/surgical ward	242 (47.0)
Operating room	186 (36.9)
Intensive care unit	76 (15.1)
Other	11 (2.1)
**Discharge disposition**	
Expired	6 (1.2)
Home	398 (77.3)
Subacute nursing facility or rehabilitation	91 (17.7)
Other	20 (3.9)
**Top 5 ICD-10 principal diagnosis categories**	
Musculoskeletal and connective tissues diseases	125 (24.8)
Circulatory system diseases	82 (16.3)
Infectious diseases	62 (12.3)
Trauma, injury, and poisoning	37 (7.3)
Digestive system diseases	28 (5.6)

*Tabled values are: mean ± standard deviation; median (interquartile range); or number (percent)

#### CTT analyses

For the 12 new CR items, there were no sparse (n < 10) response categories. Cronbach's alpha = 0.97; adjusted item-total score correlations ranged from 0.72–0.91. For the nine existing and three new PR items, there were no sparse response categories. Cronbach’s alpha = 0.96; adjusted item-total score correlations ranged from 0.71–0.86. CTT analyses indicated the appropriateness of proceeding toward creating total scores.

#### Summed score distribution

For CR and PR items, minimum/maximum possible summed scores were, respectively, 12 and 60. For CR items, mean inpatient summed score was 37.1 (SD = 12.4; median = 38.0). For PR items, mean inpatient summed score was 34.7 (SD = 14.7; median = 33.0). In both item sets, minimum/maximum possible summed scores were observed. For CR items, there were no significant floor/ceiling effects: *n* = 9 (1.7%) had score = 12; *n* = 18 (3.5%) had score = 60. The CR score distribution appeared largely normal (skewness =  − 0.1; excess kurtosis =  − 0.7). For PR items, there were no significant floor/ceiling effects: *n* = 22 (4.3%) had score = 12; *n* = 24 (4.7%) had score = 60. The PR score distribution had normal skewness (0.2), with minor excess kurtosis (− 1.2). Summed score histograms are displayed in Online Appendix Figures 1–2.

Pearson correlation between CR and PR summed scores was moderately high (0.74). We considered this magnitude to reflect similarity in constructs assessed, balanced by their independent assessment tasks and respondent perspectives (clinician vs. patient). The correlation offered initial evidence of item set validity and supported the appropriateness of linking CR to PR items.

#### CCFA

CR model: All factor loadings were ≥ 0.50 (indicating item construct validity); no residual correlations were > 0.20 (indicating item local independence). Model fit indices were: CFI, TLI = 0.99, RMSEA = 0.18, SRMR = 0.07. PR model: All factor loadings were ≥ 0.50; no residual correlations were > 0.20. Model fit indices were: CFI, TLI = 0.98, RMSEA = 0.14, SRMR = 0.06.

#### CBFA

Because RMSEA values exceeded criterion, we conducted CBFA to determine if item sets were essentially unidimensionality and total scores sufficiently reliable to recommend their use. For CR items, omega = 0.99, omega-H = 0.95, and 95% of total score reliable variance was general factor-attributable (omega-H/omega ratio = 0.95); therefore, CR total score is recommended for use. For PR items we also recommend total score use: omega = 0.99, omega-H = 0.95, and 96% of total score reliable variance was general factor-attributable (omega-H/omega ratio = 0.96).

#### DIF

No CR or PR items in age or gender factor studies were flagged for DIF; thus, no “score impact” analyses were required.

### Objective 3: link new items to PROMIS PF

#### Link 12 new CR items to nine existing PR items

Pearson and disattenuated summed score correlations were sufficiently high (0.73 and 0.77, respectively) to justify IRT-based linking [36, 37]. We anchored PR item parameters at PF bank values and calibrated, via FPC, CR items, putting their parameters on the PF metric.

#### Three new PR items to nine existing PR items

Summed score Pearson and disattenuated correlations were sufficiently high (0.90 and 0.98, respectively) to justify IRT-based linking. We again anchored the nine PR parameters at PF bank values and calibrated new PR items, placing their parameters on the PF metric.

### Objective 4: construct SF measures﻿

We reduced the length of 12-item PF assessments, first selecting five CR items optimizing clinical relevance and score-level precision (Table 2). The content-parallel set of five PR items was then evaluated for relevance and precision prior to acceptance. CR and PR 5-item SF measures thus contain the most clinically relevant assessment items providing best attainable score-level reliabilities across our targeted measurement range.

#### SF score distribution﻿

**Fig. 1:**
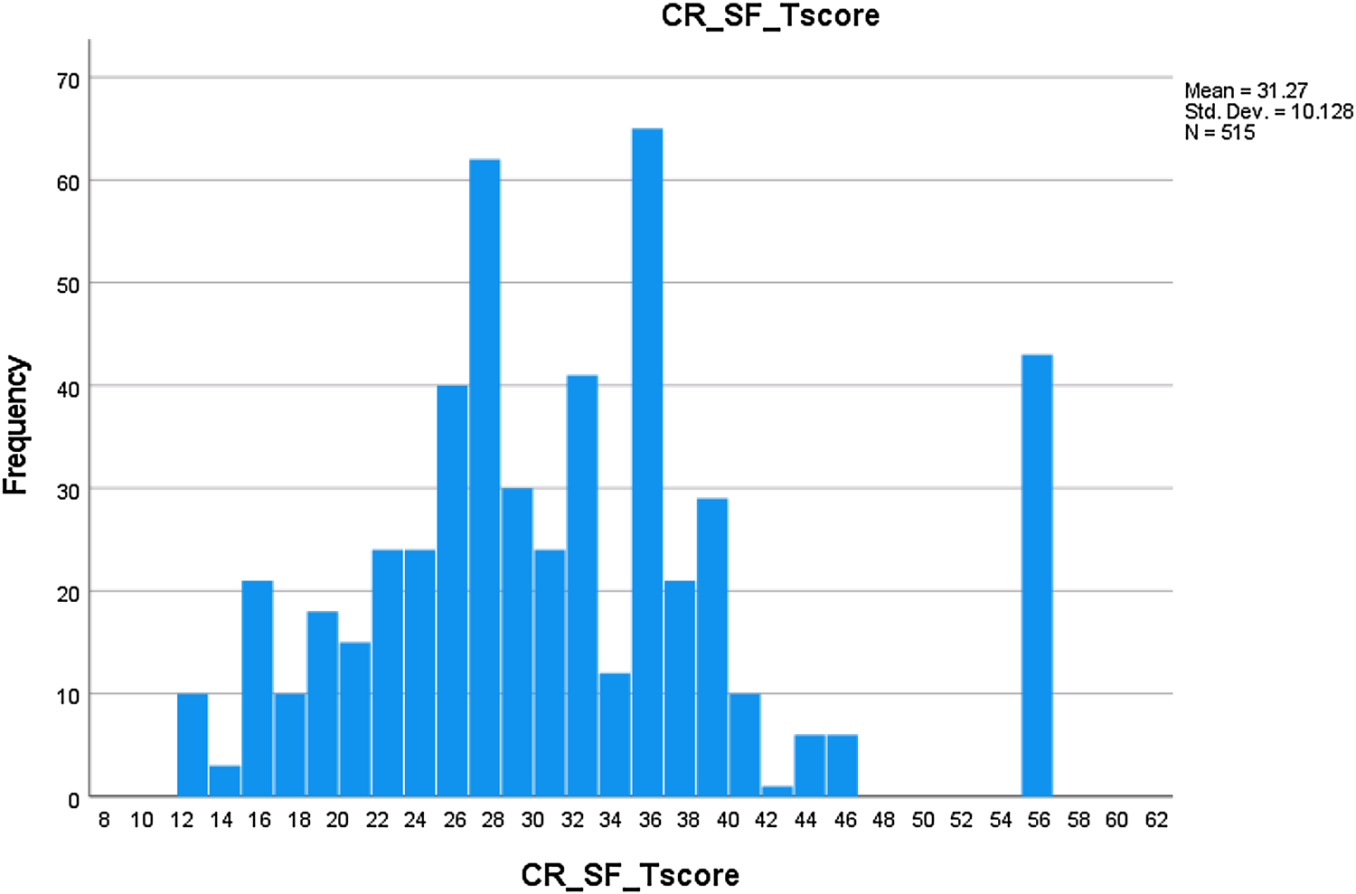
CR SF T-score distribution

**Fig. 2:**
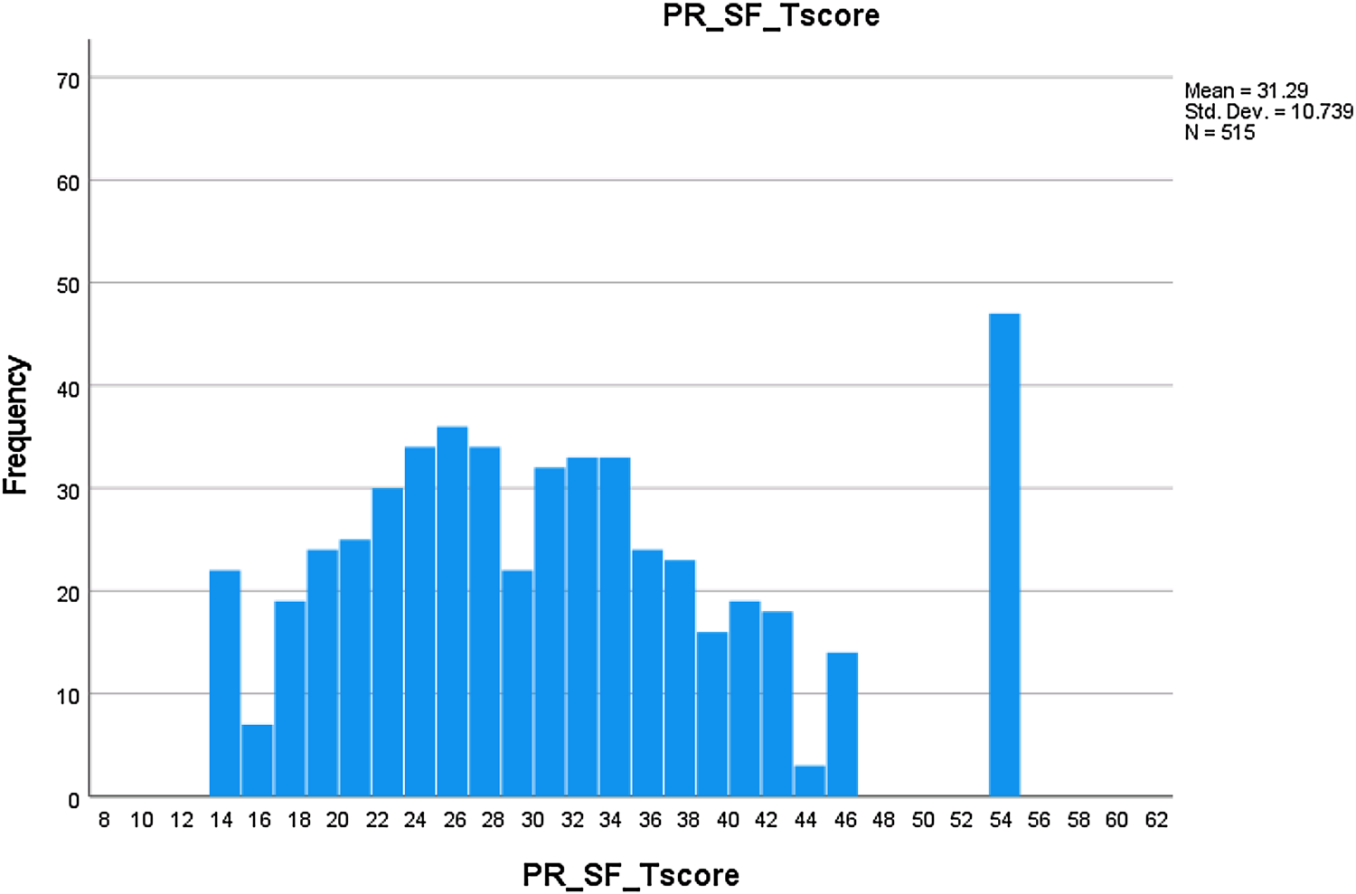
PR SF T-score distribution

SF T-score distributions are displayed in Figs. 1–2. For the CR SF, minimum/maximum observed T-scores were 12.4 and 55.1, respectively; mean score was 31.3 (SD = 10.1; median = 30.0). Distribution skewness = 0.70, excess kurtosis = 0.46 (essential normality). *N* = 10 (1.9%) had the minimum possible score = 12.4; *n* = 43 (8.3%) had the maximum possible score = 55.1 (no significant floor/ceiling effects).

For the PR SF, minimum/maximum observed T-scores were 13.8 and 54.3, respectively; mean score was 31.3 (SD = 10.7; median = 30.2). Distribution skewness = 0.58, excess kurtosis =  − 0.24 (essential normality). *N* = 22 (4.3%) had the minimum possible score = 13.8; *n* = 47 (9.1%) had the maximum possible score = 54.3 (no significant floor/ceiling effects).

#### SF correlation and reliability

**Fig. 3:**
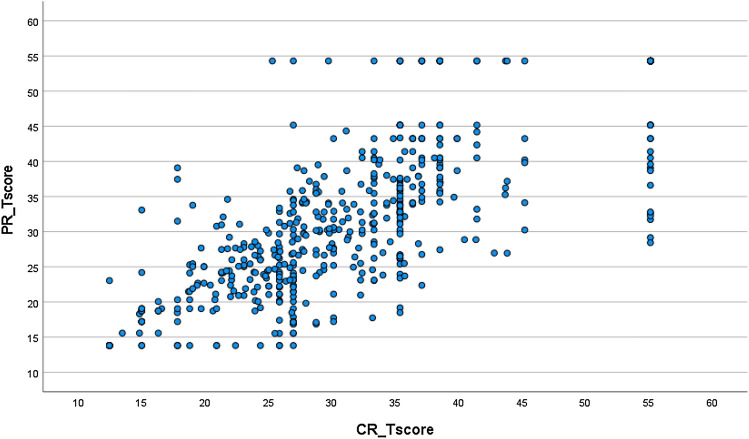
CR and PR T-score scatter plot

**Fig. 4:**
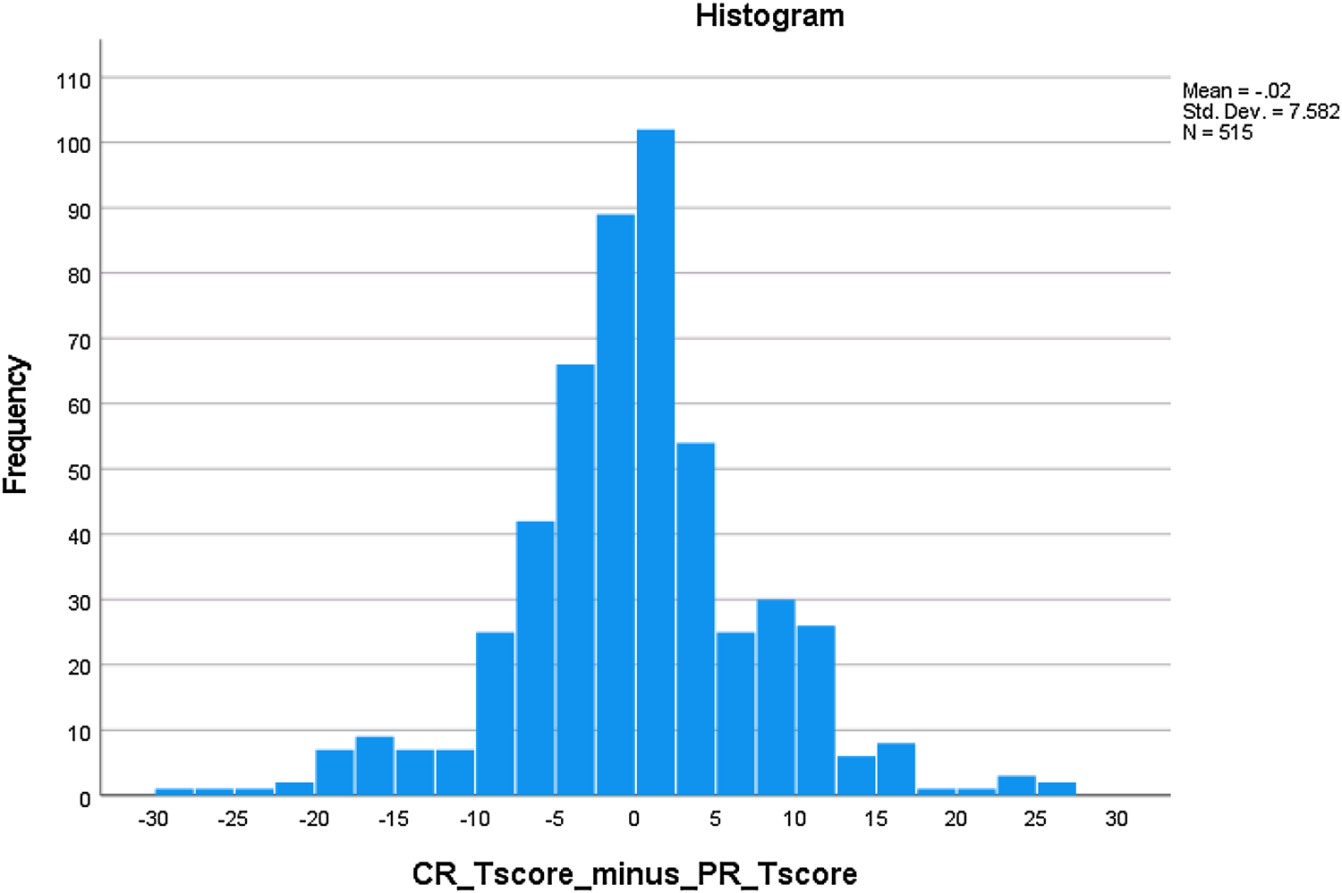
CR and PR T-score differences distribution

Pearson correlation between CR and PR SF scores was 0.74, a substantial magnitude but not enough to conclude one SF’s scores were interchangeable with or redundant in regards to the other’s: Each SF contributed related but conceptually distinct inpatient PF information. In the CR-PR score scatter plot (Fig. 3), typical score pairs appear relatively well-aligned, while less-well-aligned score pairs seem often associated with extreme scores. Where clinicians had assigned patients the highest possible score, those patients have reported more variable (inevitably lower) PF status; where patients had assigned themselves the highest possible score, clinicians have documented more variable (often lower) PF. Because each score in a score pair represents a unique source of information (clinician vs. inpatient), such score differences create opportunities for patient-clinician discussion and reconciliation. A histogram depicts the clinician vs. patient score difference distribution (Fig. 4). Differences were normally distributed: 63.7% within + / − 0.5 SDs, 85.8% within + / − 1 SD.

**Fig. 5:**
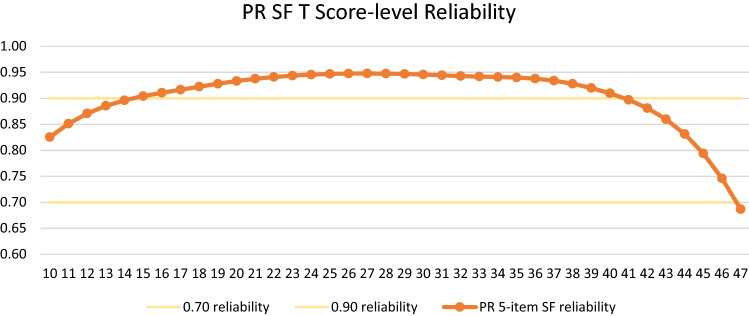
PR SF expected score-level reliability

For the CR SF, T-scores from 13–45 had score-level reliabilities ≥ 0.90 (Online Appendix Figure 3); SE-based reliability = 0.94. For the PR SF, T-scores from 14–41 had score-level reliabilities ≥ 0.90 (Fig. 5); SE-based reliability = 0.93. Thus, CR and PR SFs exhibited good-to-excellent score-level reliabilities across our targeted T-score range.

#### SF summed score conversion tables

We created scoring conversion tables, based on each SF’s item parameters, which indicate the most appropriate T-score and associated SE for each possible summed score (Online Appendix Tables 1–2).

## Discussion

Two item content-matched, precise, score-level targeted 5-item SFs were developed for clinician and patient reporting of inpatient PF: “PROMIS Physical Function-5” (PF-5) CR and PR assessments. They are effective, efficient PF assessments, offering complementary perspectives on PF status to guide treatment and decision making for acutely ill hospitalized patients.

We identified four strengths of the PF-5 assessments. First, they help close a gap in inpatient-setting PF reporting. While many instruments have been developed to assess PF, few are usable for tracking and understanding PF across health conditions, care settings, and time. Pertaining to hospitalization, few tools are easily applied to capture and track pre-morbid, intra-hospital, and post-discharge PF. Additionally, many measures are strictly patient- or clinician-reported, limiting broad application and the collecting of holistic perspectives of PF [38]. Using PF instruments normed to different scales across the peri-hospital period inhibits understanding PF improvement or decline trajectories, critical in guiding clinician decisions regarding safe mobilization techniques, use of PT and rehabilitation services, and discharge planning across a patient population. The PF-5 assessments create opportunities for capturing patient trajectories in and out of hospital, providing two SF versions of PROMIS PF specifically designed to meet hospital settings’ unique clinical needs and operational realities.

Second, while PF-5 CR and PR are usable separately, when operationally feasible using them in concert, gathering information from multiple perspectives, can create a more holistic picture of PF. Dual-perspective assessments could assist care teams identifying and addressing discrepancies in perceptions of patient level of independence or need for assistance to complete functional tasks at key points in time (e.g., hospital discharge). In practice, this information could elucidate reasoning behind patient/family concerns about a discharge plan if patient perceptions differ significantly from the clinical team’s. Third, having CR and PR assessment versions maximizes the care team’s ability to capture the PF information they need to make decisions during hospitalization. While clinicians cannot directly assess patients’ pre-morbid PF, patients can self-report that information upon admission. Pre-morbid function assessment is not a replacement for clinician assessment, but it is likely the best alternative available. Both versions norm to a common scale/metric, helping the care team synthesize differently-sourced information.

Fourth, with simple and direct assessment items, PF-5 tools can support a shared language and understanding between diverse clinicians and patients/families about differences in patient PF over time. Multiple PF assessment tools exist; few are widely familiar to practitioners across diverse specialties and healthcare settings; even fewer are understood by all care team members, including patients/families.

PF-5 items represent content our clinical team (physicians, nurses, PTs) felt was most relevant and feasible for assessing PF in the acute care setting. Thus, PF-5 assessment focuses on the PF continuum’s lower range. While this is a limitation, if considering the PF-5 for all settings and conditions, we do not believe it a significant limitation for its successful use in the peri-hospitalization period. Higher PF levels are infrequently assessed in the hospital setting: Primarily, because exemplar activities are most often not required for safe discharge home; secondarily, because they are operationally impractical.

Stair climbing activity is not included in final item sets. While PTs are trained to assess stair climbing, other care team members are typically not. Furthermore, not all patients require stair climbing ability in their daily lives; therefore, it is not a condition for safe discharge home for all patients. If stair climbing or other less common functional tasks are required for discharge, PTs will need to assess such abilities.

There are several limitations to our findings. First, our patient sample size was relatively small; however, it was adequate to robustly evaluate, calibrate, and link clinician and patient item responses. Second, we used a convenience sampling strategy based on routine PT practice patterns. Patients seen during PT evaluation do comprise a representative subset of all inpatients with increased physical limitations. Still, PF-5 items may be limited in detecting PF gradations significantly exceeding population averages, constraining their utility among high-functioning inpatients. Further PF assessments with the PF-5 in a broader hospital population are needed. Third, our sample of participating PTs represents a convenience sample: PTs volunteered to participate, requiring them to include an additional clinician-reported patient assessment into their existing clinical work flow. The actual population of KP PTs is in the 100 s, rather than 1000 s, of possible participants, given our project’s location-specific nature. Thus, the number of PTs sampled is a healthy proportion of practicing PTs available. Note these *N* = 36 PTs provided *N* = 515 assessments, which became our dataset of clinician-reported evaluations. Fourth, assessing PF is a standard skill for PTs; however, other clinician groups (nurses, physicians, care coordinators) using the PF-5 may differ in assessment ability. During this study, PTs were best able to integrate testing of the broader set of assessment items into their clinical workflow. Fifth, we did not evaluate PT evaluation timing relative to hospital discharge, which may be relevant to acutely ill patients displaying dynamic PF over a single hospitalization.

For future directions, next steps in measure development should include systematic estimates of test–retest reliability, responsiveness, and within and between “rater” (clinician) agreement. For rater agreement analyses, we plan to obtain a minimum *n* = 30 assessments per rater to conduct defensible analyses with robust findings. There are also feasibility “next steps.” In this project, patients and PTs each completed 12 candidate items, generally taking some 15 min to complete the full assessment process. We were unable to assess acceptability of final PF-5 CR/PR versions; however, we expect PF-5 completion to be quite brief, particularly once integrated into our EHR. Future work will study PF-5 uptake among practitioners, especially nursing and inpatient care team members; it will also assess timing and logistical characteristics.

The PF-5 measures are linked to the PROMIS PF metric, enabling CR and PR assessments to be used to evaluate more complete patient PF trajectories. Together, these measures streamline information gathering and, in conjunction with the broader set of available PROMIS PF assessments, make standardized PF assessment attainable across inpatient, outpatient, home-based, and virtual settings. This improved ability to track and understand PF in the peri-hospitalization period should create a watershed of insights for developing and applying targeted interventions aimed at sustaining or improving PF at critical junctures in patients’ lives.

## Supplementary Information

Below is the link to the electronic supplementary material.

Supplementary file1 (DOCX 44 kb)

Supplementary file2 (DOCX 43 kb)

Supplementary file3 (DOCX 38 kb)

Supplementary file4 (DOCX 33 kb)

Supplementary file5 (DOCX 33 kb)

## Abbreviations

CBFA: Confirmatory bifactor analysis
CCFA: Categorical confirmatory factor analysis
CFI: Confirmatory fit index
CR: Clinician-report
CTT: Classical test theory
DIF: Differential item functioning
EHR: Electronic health record
IRT: Item response theory
KP: Kaiser Permanente
NU: Northwestern University
PF: Physical function
PR: Patient-report
PROMIS: Patient-Reported Outcomes Measurement Information System
PT: Physical therapist
RMSD: Root mean square difference
RMSEA: Root mean square error of approximation
SD: Standard deviation
SE: Standard error
SF: Short form
SRMR: Standardized root mean residual
TLI: Tucker–Lewis index
WLSMV: Weighted least square-mean and variance adjusted

## Declarations

### Data availability

De-identified data may be requested from Kaiser Permanente, which retains data storage and stewardship responsibilities. Data requests are subject to review by Kaiser Permanente.
