# Supplementary figures and images for: New complementary perspectives for inpatient physical function assessment: matched clinician-report and patient-report short form measures from the PROMIS adult physical function item bank
Source: Qual Life Res. 2022 Mar 8;31(7):2201–12. doi: 10.1007/s11136-022-03089-z (PMC9188510; doi:10.1007/s11136-022-03089-z)

Appendix Figure 1. CR Summed Score Distribution


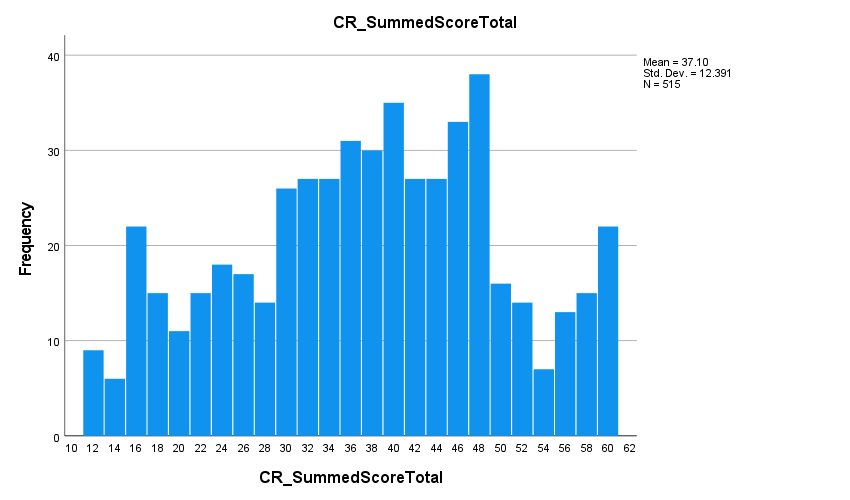

Supplement: Supplementary file 1 — Supplementary file1 (DOCX 44 kb) [file 11136_2022_3089_MOESM1_ESM.docx]

Appendix Figure 2. PR Summed Score Distribution


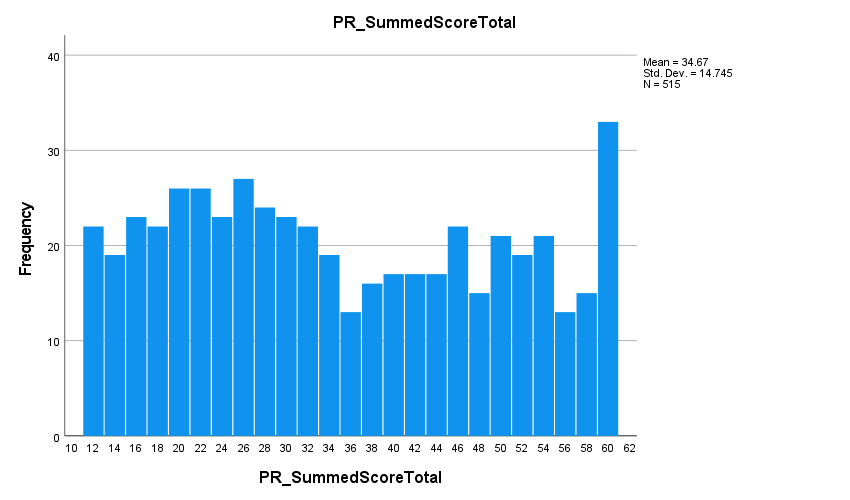

Supplement: Supplementary file 2 — Supplementary file2 (DOCX 43 kb) [file 11136_2022_3089_MOESM2_ESM.docx]
