# Supplementary material for: New complementary perspectives for inpatient physical function assessment: matched clinician-report and patient-report short form measures from the PROMIS adult physical function item bank
Source: Qual Life Res. 2022 Mar 8;31(7):2201–12. doi: 10.1007/s11136-022-03089-z (PMC9188510; doi:10.1007/s11136-022-03089-z)
Supplement: Supplementary file 3 — Supplementary file3 (DOCX 38 kb) [file 11136_2022_3089_MOESM3_ESM.docx]

Appendix Figure 3. CR SF Expected Score-level Reliability
