# Supplementary material for: New complementary perspectives for inpatient physical function assessment: matched clinician-report and patient-report short form measures from the PROMIS adult physical function item bank
Source: Qual Life Res. 2022 Mar 8;31(7):2201–12. doi: 10.1007/s11136-022-03089-z (PMC9188510; doi:10.1007/s11136-022-03089-z)
Supplement: Supplementary file 4 — Supplementary file4 (DOCX 33 kb) [file 11136_2022_3089_MOESM4_ESM.docx]

Appendix Table 1. **PROMIS Clinician-reported Inpatient Physical Function Short Form (CR PF-5):**

**Raw Summed Score to T score Lookup Table**

| **Raw Summed Score** | **T score** | **SE *** |
| --- | --- | --- |
| 5 | 12.45 | 2.05 |
| 6 | 13.74 | 2.35 |
| 7 | 15.32 | 2.43 |
| 8 | 17.29 | 2.30 |
| 9 | 18.97 | 2.10 |
| 10 | 20.41 | 2.01 |
| 11 | 21.79 | 2.00 |
| 12 | 23.16 | 2.04 |
| 13 | 24.55 | 2.07 |
| 14 | 25.87 | 2.07 |
| 15 | 27.17 | 2.10 |
| 16 | 28.55 | 2.16 |
| 17 | 30.08 | 2.28 |
| 18 | 31.75 | 2.42 |
| 19 | 33.52 | 2.53 |
| 20 | 35.34 | 2.55 |
| 21 | 37.20 | 2.50 |
| 22 | 39.23 | 2.44 |
| 23 | 41.84 | 2.53 |
| 24 | 45.15 | 3.26 |
| 25 | 55.14 | 7.19 |

*** SE = T score standard error**
