# Supplementary material for: New complementary perspectives for inpatient physical function assessment: matched clinician-report and patient-report short form measures from the PROMIS adult physical function item bank
Source: Qual Life Res. 2022 Mar 8;31(7):2201–12. doi: 10.1007/s11136-022-03089-z (PMC9188510; doi:10.1007/s11136-022-03089-z)
Supplement: Supplementary file 5 — Supplementary file5 (DOCX 33 kb) [file 11136_2022_3089_MOESM5_ESM.docx]

Appendix Table 2. **PROMIS Patient-reported Inpatient Physical Function Short Form (PR PF-5):**

**Raw Summed Score to T score Lookup Table**

| **Raw Summed Score** | **T score** | **SE *** |
| --- | --- | --- |
| 5 | 13.81 | 2.63 |
| 6 | 15.80 | 2.88 |
| 7 | 17.66 | 2.87 |
| 8 | 19.29 | 2.77 |
| 9 | 20.77 | 2.68 |
| 10 | 22.13 | 2.59 |
| 11 | 23.42 | 2.53 |
| 12 | 24.64 | 2.49 |
| 13 | 25.83 | 2.47 |
| 14 | 27.00 | 2.46 |
| 15 | 28.19 | 2.47 |
| 16 | 29.39 | 2.50 |
| 17 | 30.63 | 2.53 |
| 18 | 31.93 | 2.57 |
| 19 | 33.30 | 2.64 |
| 20 | 34.78 | 2.76 |
| 21 | 36.49 | 3.01 |
| 22 | 38.46 | 3.34 |
| 23 | 40.74 | 3.66 |
| 24 | 43.90 | 4.22 |
| 25 | 54.30 | 7.62 |

*** SE = T score standard error**
